# Pentad: a tool for distance-dependent analysis of Hi-C interactions within and between chromatin compartments

**DOI:** 10.1186/s12859-022-04654-6

**Published:** 2022-04-02

**Authors:** Mikhail D. Magnitov, Azat K. Garaev, Alexander V. Tyakht, Sergey V. Ulianov, Sergey V. Razin

**Affiliations:** 1 Institute of Gene Biology, Russian Academy of Sciences, Moscow, Russia 119334; 2 Faculty of Biology, Lomonosov Moscow State University, Moscow, Russia 119234

**Keywords:** 3D genome, Chromosome conformation capture, Hi-C, Chromosomal compartments, Genomic interactions, Hi-C data analysis

## Abstract

**Background:**

Understanding the role of various factors in 3D genome organization is essential to determine their impact on shaping large-scale chromatin units such as euchromatin (A) and heterochromatin (B) compartments. At this level, chromatin compaction is extensively modulated when transcription and epigenetic profiles change upon cell differentiation and response to various external impacts. However, detailed analysis of chromatin contact patterns within and between compartments is complicated because of a lack of suitable computational methods.

**Results:**

We developed a tool, Pentad, to perform calculation, visualisation and quantitative analysis of the average chromatin compartment from the Hi-C matrices in *cis*, *trans*, and specified genomic distances. As we demonstrated by applying Pentad to publicly available Hi-C datasets, it helps to reliably detect redistribution of contact frequency in the chromatin compartments and assess alterations in the compartment strength.

**Conclusions:**

Pentad is a simple tool for the analysis of changes in chromatin compartmentalization in various biological conditions. Pentad is freely available at https://github.com/magnitov/pentad.

**Supplementary Information:**

The online version contains supplementary material available at 10.1186/s12859-022-04654-6.

## Background

High-throughput chromosome conformation capture (Hi-C) studies of the 3D genome architecture have revealed several features of spatial genome organization in higher eukaryotes. Within the chromosome territories [1], transcriptionally active and repressed loci are spatially segregated into A and B compartments [2], that closely resemble eu- and heterochromatin, respectively. At the scale of megabases, chromatin is folded into topologically associated domains (TADs) [3, 4], commonly interpreted as relatively stable globules. In mammals, TAD boundaries are enriched in CTCF/cohesin binding [3] and demarcate areas of enhancer action [5]. Regulatory elements within TADs, such as promoters and enhancers, interact with each other and form chromatin loops, whose bases are frequently marked with binding of architectural proteins such as CTCF [6], YY1 [7], ZNF143 [8], and others [9, 10]. As revealed by the depletion of subunits of the cohesin complex [11] and CTCF [12], the overwhelming majority of TADs and loops in mammalian cells are established by cohesin-driven CTCF-restricted chromatin fiber extrusion. In contrast, mechanisms of compartment formation and maintenance are largely unknown. Compartment profile along the genome and contact patterns within A/B compartments are sensitive to changes in gene expression during cell differentiation [13] and cell senescence [14, 15], alter in response to osmotic stress [16] and depend on the activity of loop extrusion machinery [17, 18]. Despite the increasing number of observations on dynamics of compartment structure in different biological conditions, the determinants of genome compartmentalization remain elusive [19]. Thus, multiple ongoing studies aim to shed light on the aspects of compartment formation [20].

In contrast to TAD and loop annotation and visualization tools (Additional file 1: Table S1), only a limited number of methods for A/B compartments annotation and analysis are available. For instance, compartments were initially discovered using principal component analysis (PCA) [2] which became a method of choice for compartment annotation. Recently, CscoreTool [21] and POSSUMM [22] were reported as a PCA-based memory-efficient algorithms for compartment annotation, while SNIPER [23] and Calder [24] algorithms were developed for sub-compartment detection in moderately covered Hi-C data and at various map resolutions, respectively. However, averaged contact frequency between genomic bins belonging to different compartments is mostly analysed using the saddle plot diagram [25, 26]. Despite its utility, saddle plot representation is clearly lacking the separation of short- and long-range interactions, and is not convenient to analyze the average contact frequencies at a predefined scale. Thus, the available tools cannot systematically probe the dynamics and perturbations of chromatin contact patterns within compartments. To fill this gap, we developed a new tool, Pentad, which can calculate, visualize and quantify the average compartment structure within a predefined range of genomic distances. Using published Hi-C datasets, we demonstrate that Pentad accurately detects the redistribution of contacts between and within A and B compartments without requiring additional analyses.

## Implementation

**Fig. 1:**
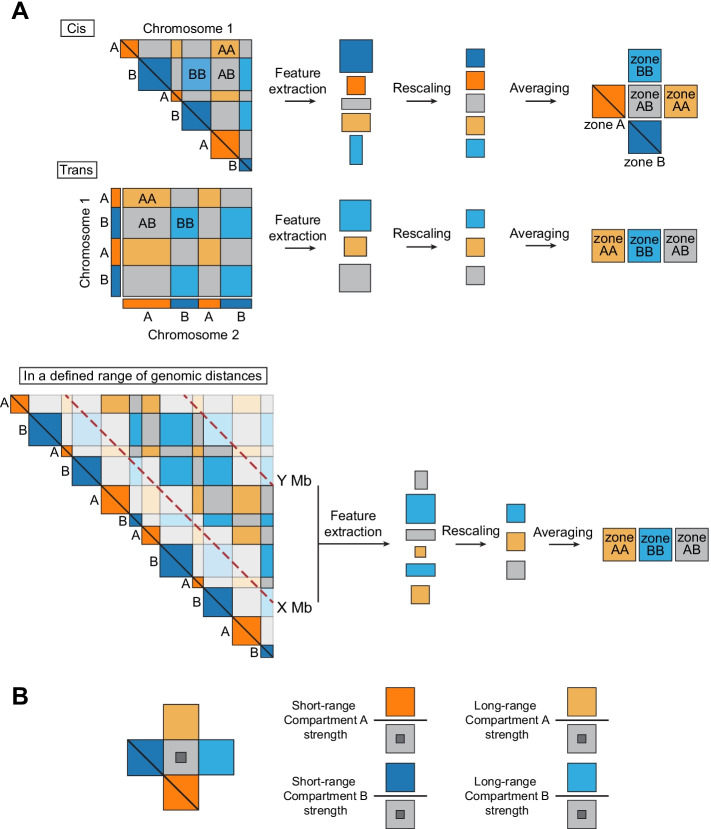
Pentad pipeline for average compartment and compartment strength calculations. **A** Schematic representation of the Pentad pipeline for averaging compartments in *cis*, *trans*, and *cis*-by-distance. **B** Schematic representation of the compartment strength calculation by Pentad

The average compartment visualisation provided by Pentad represents short- and long-range contacts within A and B compartments together with intercompartmental interactions. The visualisation comprises several types of areas from the Hi-C matrix that are determined based on the annotated A/B compartment signal, which is usually a first principal component (PC1) from PCA of the Hi-C matrix (Fig. 1A). The obtained visualisation is then used to estimate the average compartment strength.

To create an average compartment visualisation, compartment areas of different types are extracted from the observed-over-expected Hi-C matrix and subjected to filtering. First, areas are filtered based on their dimensions in genomic bins, because small areas are likely to represent noisy regions of the Hi-C matrix. Next, areas with a low number of contacts are removed because of their poor resolution. Finally, areas at a distance between the anchors larger than a specific cutoff value are removed. Areas that meet the criteria are then rescaled using bilinear interpolation into squares of a predefined size. Rescaled areas of the same type are averaged genome wide using median pixel values, and they are aggregated into one plot.

To calculate compartment strength, the mean value of contacts from areas representing interactions within A and B compartments are divided by the mean value of contacts between these compartments (Fig. 1B). To avoid bias towards low values of the compartment signal when estimating intercompartment interactions, the edges of the corresponding average compartment square are cropped to remove residual interactions occurring in the A and B compartments. Compartment strength is calculated for each chromosome from the Hi-C matrix, enabling a comparison of the results with statistical tests.

Current implementation of Pentad is provided as a set of Python scripts that can average *cis* and *trans* Hi-C interactions, to stratify the compartment areas by genomic distance, and calculate compartment strength directly from the average compartments (see Additional file 1: Methods and Additional file 1: Figure S1 for more details). The required input files are a Hi-C matrix in *cooler* format [27] and a compartment signal in the *bedGraph* format.

## Results

**Fig. 2:**
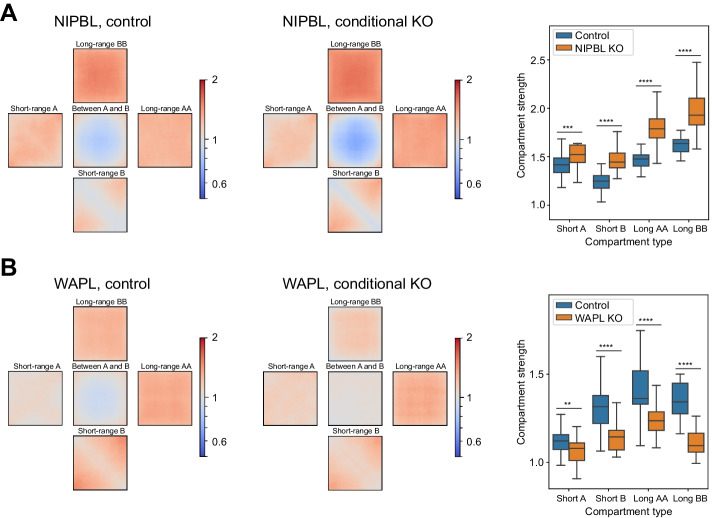
Pentad accurately calculates averaged compartments and detects their dynamics. **A** *Cis*-pentads for mouse cells with conditional knock-out (KO) of cohesin loading factor (NIPBL; data from Schwarzer et al. [18]). **B** *Cis*-pentads for human cells with knock-out (KO) of cohesin releasing factor (WAPL; data from Haarhuis et al. [17]). Quantification of compartment strength for different types of compartment interactions is shown to the right with box plots. **, *P-*value ≤ 0.01; ***, *P-*value ≤ 0.001; ****, *P-*value ≤ 0.0001, Wilcoxon signed-rank test

To demonstrate the utility of the Pentad algorithm, it was first applied to the Hi-C datasets with a known impact on the compartment’s structure. Thus, we focused on conditional knock-outs of cohesin loading factor NIPBL [18] and cohesin release factor WAPL [17] in mammalian cells. As previously reported, removing NIPBL enhances chromatin compartmentalization, and knocking out WAPL compromises the segregation of A and B compartments. We confirmed the increase in compartment segregation in NIPBL-deficient cells (Fig. 2A, the central square of the average compartment), and we found that both A and B compartments gain interactions at long genomic distances. In addition, we showed that increased compaction of the A compartment is provided by a shift of the interactions from the main diagonal of the Hi-C matrix to longer distances because of the disruption of TADs. In WAPL-deficient cells (Fig. 2B), we observed decreased compartment segregation, with the B compartment losing interactions at all genomic distances and the A compartment losing interactions only on long-range distances. We also observed a gain of contacts at short genomic distances for the A compartment, potentially caused by an increased number of loops upon WAPL knock-out.

**Fig. 3:**
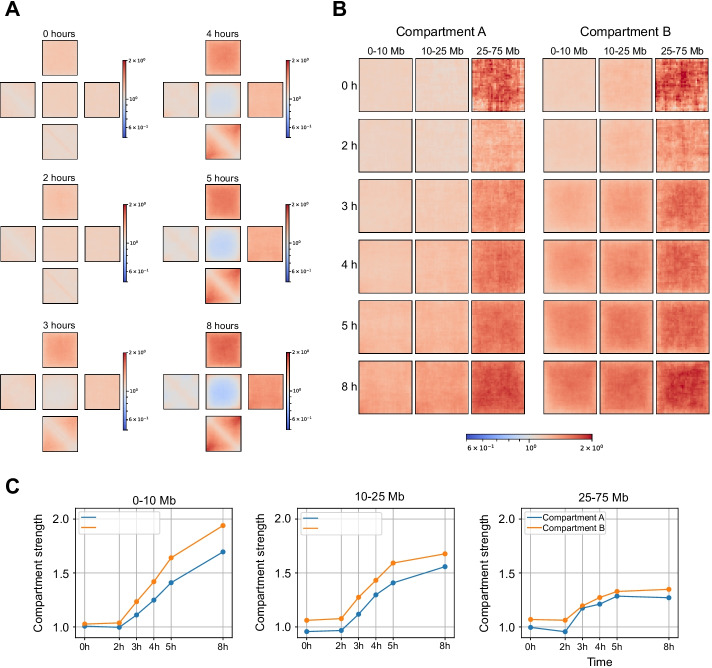
Pentad analysis of compartment dynamics upon release of cells from prometaphase arrest. **A** *Cis*-pentads for time points after release from prometaphase arrest. **B** *Cis*-by-distance-pentads at time points after release from prometaphase arrest. **C** Quantification of compartment strength

**Fig. 4:**
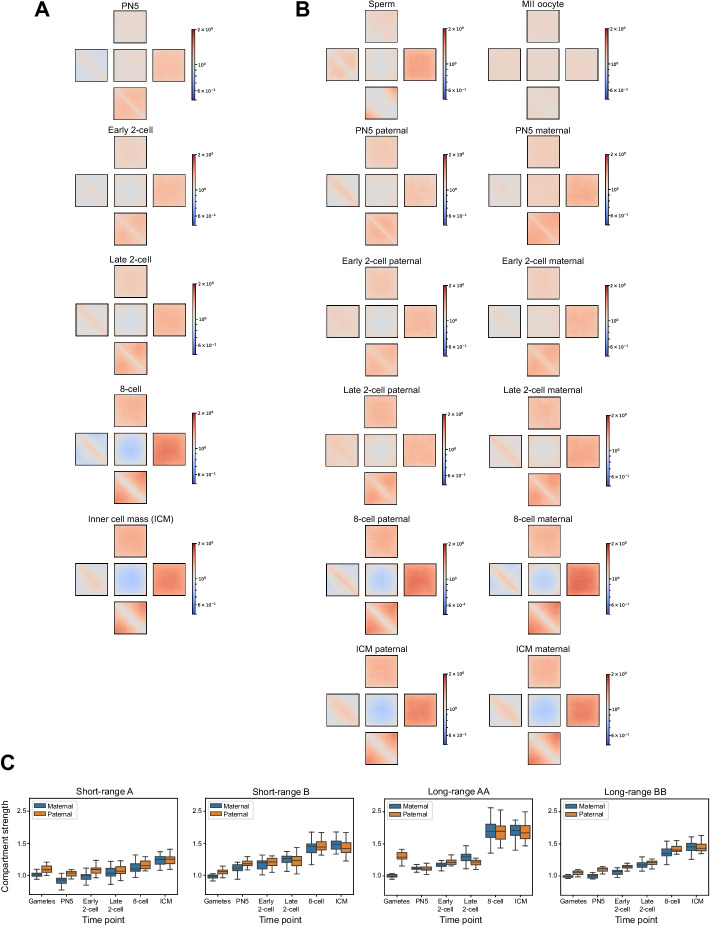
Pentad analysis of compartment dynamics during embryonic development. **A** *Cis*-pentads for several consecutive stages of mouse embryo development: PN5 zygotes, 2- and 8-cell embryos and inner cell mass from blastocysts (ICM). **B** *Cis*-pentads for gametes (sperm and oocytes arrested in metaphase of meiosis II) and developing mouse embryos constructed for maternal and paternal genomes separately. **C** Quantification of compartment strength for different types of compartment interactions

We next applied Pentad to a time-course datasets to assess its ability to capture the A/B compartment dynamics. First, we probed the compartmentalization that occurs when human cells transition from mitosis to G1 [28]. As expected, in the prometaphase and at the entry of G1, we did not see any compartment structure. It emerges 3 h after the release of the cells from prometaphase arrest (Fig. 3A). When applied to the compartments stratified by genomic distance, Pentad revealed that A and B compartments have different assembly kinetics at short and long distances (Figs. 3B, 3C). Second, we inspected changes in compartmentalization during the early development of mouse embryos [29]. Here, we observed a prolonged formation of chromatin compartments, which are reduced after fertilisation and re-established during preimplantation development (Fig. 4A). By analysing allele-specific Hi-C contact matrices, we detected that compartmentalization already occurs in zygotes for the paternal genome, but it is weakly pronounced until the later stages for the maternal genome for short-range A and long-range B compartments (Figs. 4B, 4C).

## Conclusions

Pentad is a simple tool that allows one to analyse chromatin compartments based on a Hi-C matrix and compartment signal only. Our results demonstrate the tool’s utility for quantitative analysis of A/B compartments and tracing the changes of the average compartment structure at different genomic scales in various biological conditions. It is fast and easy to use, and it provides reliable results, and this makes Pentad a useful tool for analysing the impact of various factors on the 3D genome organization. We anticipate that Pentad could simplify data interpretation and stimulate formulating novel hypotheses to understand the mechanisms underlying chromatin compartments formation, and would be used for the analysis of A/B compartment structure in a wide range of biological conditions and model systems.

### Availability and requirements

Project name: Pentad.

Project home page: https://github.com/magnitov/pentad.

Operating system(s): Platform independent.

Programming language: Python.

Other requirements: conda.

License: MIT License.

Any restrictions to use by non-academics: None.

## Supplementary Information

**Additional file 1.** Supplementary Information: Supplementary Methods, Figure S1 with Pentad technical performance assessment, and Table S1 with a list of available tools for the pile-up analysis of Hi-C features.

## Data Availability

The datasets re-analysed during the current study are available in the NCBI GEO repository via accession numbers GSE63525, GSE93431, GSE95014, GSE133462, GSE82185. The developed tool and code used for the analysis are available at https://github.com/magnitov/pentad.

## Abbreviations

3D: Three-dimensional
CTCF: CCCTC-binding factor
GEO: Gene Expression Omnibus
Hi-C: High-throughput chromosome conformation capture
ICM: Inner cell mass
NIPBL: Nipped-B-like protein
PCA: Principal component analysis
TAD: Topologically associated domains
WAPL: Wings apart-like protein homolog
YY1: Yin yang 1
ZNF143: Zinc finger protein 143
