# Supplementary material for: Pentad: a tool for distance-dependent analysis of Hi-C interactions within and between chromatin compartments
Source: BMC Bioinformatics. 2022 Apr 2;23:116. doi: 10.1186/s12859-022-04654-6 (PMC8976968; doi:10.1186/s12859-022-04654-6)
Supplement: Supplementary file 1 — Additional file 1. Supplementary Information: Supplementary Methods, Figure S1 with Pentad technical performance assessment, and Table S1 with a list of available tools for the pile-up analysis of Hi-C features. [file 12859_2022_4654_MOESM1_ESM.pdf]

Pentad: a tool for distance-dependent analysis of Hi-C interactions within and between chromatin compartments

Mikhail Magnitov, Azat Garaev, Alexander Tyakht, Sergey Ulianov and Sergey Razin

Additional File 1

Supplementary Methods

Pentad development

Pentad is created using the following Python packages: argparse, multiprocess, os, matplotlib, seaborn, json, cv2, numpy, pandas, cooler, cooltools, and warnings. All analyses presented in the paper were run on Ubuntu 18.04.2 x86_64-linux-gnu on 2 × Intel Xeon CPU E5-2680 @ 2.70 GHz, 192 GB RAM server in single core mode.

Pentad parameters

- **cool_file**
  Path to the cool file with Hi-C matrix for which an average compartment should be calculated.
- **comp_signal**
  Path to the bedGraph file with compartment signal.
- **rescale_size**
  Size to which all the areas of the average compartment will be rescaled. The default is 33 bins.
- **min_dimension**
  Minimum dimension of an area (in genomic bins) to be considered for average compartment calculation. It is used to filter unreliable regions with noisy compartment signal. The default is 3 bins.
- **max_zeros**
  Maximum fraction of bins with zero contacts in an area. It is used to filter out sparse regions of the Hi-C matrix. The default is 0.5.
- **cutoff**
  Maximum distance between two regions in the chromosome that form a compartment. We found that compartment regions located very far from one another don't add up any significant information, and therefore a cutoff for the distance is set. This parameter is used only for cis and cis-by-distance.
- **distances**
  Distance boundaries in Mb for cis-by-distance calculations.
- **excl_chrms**
  Chromosomes to exclude from the average compartment calculation. By default, chromosomes Y and M are excluded.
- **out_pref**
  Prefix for the output files. By default, we save the output to the same directory with prefix pentad.
- **center_width**
  Fraction of the central fragment of the average compartment that represents intercompartmental interactions used for compartment strength quantification.
- **closed**

For cis interactions stratified by distance whether to plot a closed intervals (omitting the last section).

Technical performance

Pentad's technical performance was assessed using the Hi-C dataset for the GM12878 cells (1). To assess Pentad's technical performance, the run time for different resolutions of the Hi-C matrix and different compartment sizes was computed (Figure S1). For resolution analyses, compartments were annotated for 20-kb, 50-kb, 100-kb, 200-kb, 500-kb, and 1-Mb Hi-C matrices. To analyse compartment size, compartments with average sizes of 100-kb, 200-kb, 500-kb, 1-Mb, 2-Mb, and 5-Mb were generated using BEDTools v2.25.0 (2). Pentad was run in *cis* and *trans* modes using the generated compartment signals and the same parameters as described in the section 'Analysis of average compartments'.

Processing of public Hi-C data

Published Hi-C datasets used in this study were downloaded from the GEO database via accession numbers GSE63525, GSE93431, GSE95014, GSE133462, GSE82185. For GSE93431, data from tamoxifen treated control were used. For GSE95014, data from WAPL clone 1.14 were used. For GSE133462, data from replicates 1 and 2 were pooled. When available in the format of *allValidPairs* or *hic*, the Hi-C data was converted into *cooler* format using JuicerTools v1.22.01 (https://github.com/aidenlab/juicer) (3) and hic2cool v0.7.3 (https://github.com/4dn-dcic/hic2cool) software (4).

A/B compartments were annotated using the cooltools v0.3.2 (https://github.com/open2c/cooltools) (5) call-compartments function for 100-kb resolution Hi-C matrices of the corresponding control experiments for each dataset. Per convention, the orientation of the compartment signal was selected such that it correlated positively with GC content.

Analysis of average compartments

For all average compartment calculations in the dataset, the mask of the compartment's signal from the corresponding control experiment was applied. Average compartments were calculated using the following Pentad parameters. For the average compartment in *cis*: '--rescale_size' set to 33, '--min_dimension' set to 3, '--max_zeros' set to 0.5, and '--cutoff' set to 0.75. For the average compartment in *trans*: '--rescale_size' set to 33, '--min_dimension' set to 3, and '--max_zeros' set to 0.1. For the average compartment in *cis-by-distance*: '--rescale_size' set to 33, '--min_dimension' set to 3, '--max_zeros' set to 0.5, '--cutoff' set to 0.75, and '--distances' set to 10 25 75.

Analysis of compartments strength

For all compartment strength calculations in the dataset, the mask of the compartment's signal from the corresponding control experiment was applied. The

same parameters as described in the section ‘Analysis of average compartments’ were used. The middle fraction of the square to estimate the intercompartmental interactions was set to 0.6. Statistical difference between the compartment strengths was determined by a two-sided Wilcoxon signed-rank test.

References

1. Rao SSP, Huntley MH, Durand NC, Stamenova EK, Bochkov ID, Robinson JT, et al. A 3D map of the human genome at kilobase resolution reveals principles of chromatin looping. Cell. 2014 Dec 18;159(7):1665–80.

2. Quinlan AR, Hall IM. BEDTools: a flexible suite of utilities for comparing genomic features. Bioinformatics. 2010 Mar 15;26(6):841–2.

3. Durand NC, Shamim MS, Machol I, Rao SSP, Huntley MH, Lander ES, et al. Juicer Provides a One-Click System for Analyzing Loop-Resolution Hi-C Experiments. Cell Syst. 2016 Jul;3(1):95–8.

4. Dekker J, Belmont AS, Guttman M, Leshyk VO, Lis JT, Lomvardas S, et al. The 4D nucleome project. Nature. 2017 Sep 14;549(7671):219–26.

5. Venev S, Abdennur N, Goloborodko A, Flyamer I, Fudenberg G, Nuebler J, et al. Zenodo; 2021. Available from: https://zenodo.org/record/5214125

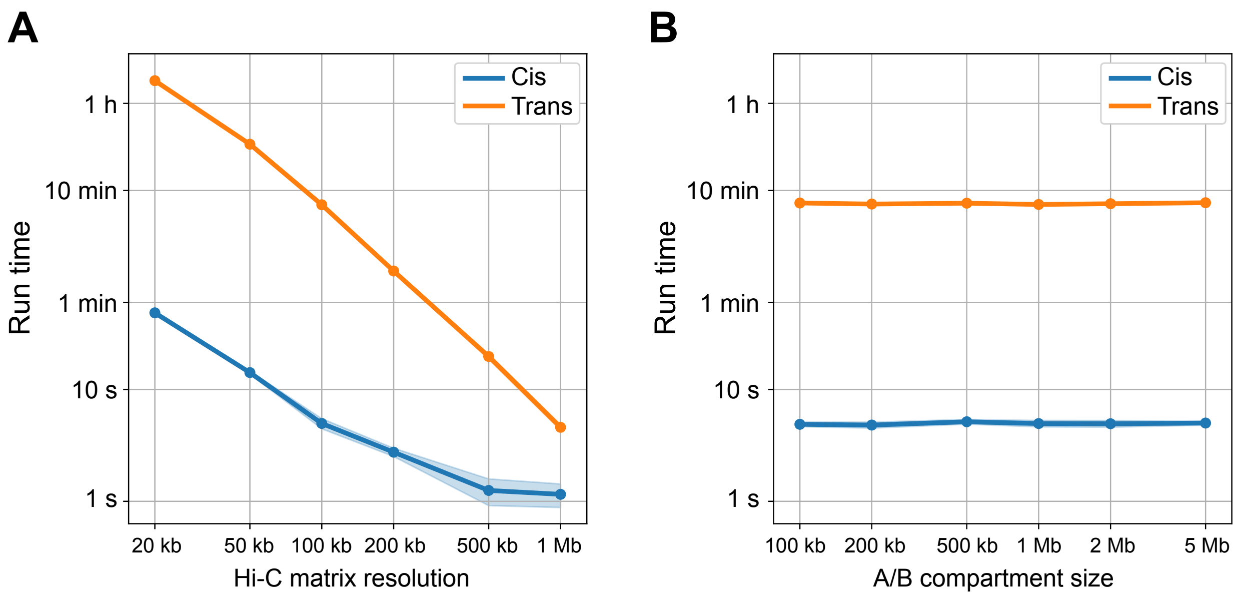

Figure S1. Pentad technical performance assessment. **(A)** Run time of Pentad *in cis* and *in trans* for different resolutions of the Hi-C matrix. **(B)** Run time of Pentad *in cis* and *in trans* for simulated compartments of different sizes and 100-kb resolution Hi-C matrix. Shadows correspond to one standard deviation from the mean run time, based on 10 consecutive Pentad executions using GM12878 Hi-C dataset from Rao et al. 2014.

	Pentad	Chromosight	coolpup.py	cooltools	FAN-C	GENOVA	HiCExplorer	HiCPeaks	Juicer	shaman
**Aggregate compartments**	+	-	-	-	-	-	-	-	-	-
**Aggregate TADs**	-	+	+	+	+	+	+	-	-	-
**Aggregate loops**	-	+	+	+	+	+	-	+	+	+
**Distance normalisation**	Expected	Expected	Expected, shifts	Expected	Expected	Expected, shifts, z-score	Expected	-	-	Custom
**API**	-	-	+	+	+	+	-	-	+	+
**CLI**	+	+	+	+	+	-	+	+	+	-
**Simple text output of pile-ups**	+	+	+	+	+	-	+	-	+	-
**Hi-C file format**	.cool	.cool	.cool	.cool	.hic, .cool	.matrix, .cool, .hic	.h5	.cool	.hic	.validPairs
**Programming language**	Python	Python	Python	Python	Python	R	Python	Python	Java	R
**Link**	https://github.com/magnitov/pentad	https://github.com/koszullab/chromosight	https://github.com/open2c/coolpuppy	https://github.com/open2c/cooltools	https://github.com/vaquerizaslab/fanc	https://github.com/robinweide/GENOVA	https://github.com/deeptools/HiCExplorer	https://github.com/XiaoTaoWang/HiCPeaks/	https://github.com/aidenlab/juicer	https://tanaylab.bitbucket.io/shaman/index.html

Table S1. List of available tools for the pile-up analysis of Hi-C features.
